# Re-calculating the cost of coccidiosis in chickens

**DOI:** 10.1186/s13567-020-00837-2

**Published:** 2020-09-14

**Authors:** Damer P. Blake, Jolene Knox, Ben Dehaeck, Ben Huntington, Thilak Rathinam, Venu Ravipati, Simeon Ayoade, Will Gilbert, Ayotunde O. Adebambo, Isa Danladi Jatau, Muthusamy Raman, Daniel Parker, Jonathan Rushton, Fiona M. Tomley

**Affiliations:** 1 Pathobiology and Population Sciences, Royal Veterinary College, Hawkshead Lane, North Mymms, AL9 7TA UK; 2 Huvepharma N.V, Uitbreidingstraat 80, 2600 Antwerp, Belgium; 3 Liverpool Science Park, Innovation Centre 2, 146 Brownlow Hill, Liverpool, L3 5RF UK; 4 Huvepharma Inc, 525 Westpark Dr, Ste 230, Peachtree City, GA 30259 USA; 5 Department of Veterinary Parasitology, College of Veterinary Science, Sri Venkateswara Veterinary University, Tirupati, Andhra Pradesh India; 6 Department of Animal Breeding and Genetics, Federal University of Agriculture, Abeokuta, Ogun State Nigeria; 7 Institute of Infection, Veterinary and Ecological Sciences, University of Liverpool, Liverpool, L69 3BX UK; 8 Department of Parasitology and Entomology, Faculty of Veterinary Medicine, Ahmadu Bello University, Zaria, Nigeria; 9 Translational Research Platform for Veterinary Biologicals, Tamil Nadu Veterinary and Animal Sciences University, Chennai, 600 051 India; 10 Slate Hall Veterinary Practice, Unit 28 Moorlands Trading Estate, Moor Lane, Metheringham, Lincolnshire, LN4 3 HX UK

**Keywords:** coccidiosis, chickens, *Eimeria*, cost, economics

## Abstract

Coccidiosis, caused by *Eimeria* species parasites, has long been recognised as an economically significant disease of chickens. As the global chicken population continues to grow, and its contribution to food security intensifies, it is increasingly important to assess the impact of diseases that compromise chicken productivity and welfare. In 1999, Williams published one of the most comprehensive estimates for the cost of coccidiosis in chickens, featuring a compartmentalised model for the costs of prophylaxis, treatment and losses, indicating a total cost in excess of £38 million in the United Kingdom (UK) in 1995. In the 25 years since this analysis the global chicken population has doubled and systems of chicken meat and egg production have advanced through improved nutrition, husbandry and selective breeding of chickens, and wider use of anticoccidial vaccines. Using data from industry representatives including veterinarians, farmers, production and health experts, we have updated the Williams model and estimate that coccidiosis in chickens cost the UK £99.2 million in 2016 (range £73.0–£125.5 million). Applying the model to data from Brazil, Egypt, Guatemala, India, New Zealand, Nigeria and the United States resulted in estimates that, when extrapolated by geographical region, indicate a global cost of ~ £10.4 billion at 2016 prices (£7.7–£13.0 billion), equivalent to £0.16/chicken produced. Understanding the economic costs of livestock diseases can be advantageous, providing baselines to evaluate the impact of different husbandry systems and interventions. The updated cost of coccidiosis in chickens will inform debates on the value of chemoprophylaxis and development of novel anticoccidial vaccines.

## Introduction

*Eimeria* are protozoan parasites that can cause the enteric disease coccidiosis in all major livestock species. The consequences of infection include malabsorption, enteritis and, in severe cases for some *Eimeria* species, mortality, compromising economic productivity and animal welfare [1]. Chickens are the most economically important hosts; more than 68 billion were farmed in 2018, representing a third of all meat produced globally in addition to 1.38 trillion eggs for human consumption [2]. Chicken production is expected to increase further in the next decade [3], highlighting the importance of pathogens that affect poultry to food security and the global agro-economy. Avian coccidiosis has previously been ranked in the top three diseases of poultry in the United Kingdom (UK) based on economic significance [4], and in the top ten veterinary diseases based on impact on the poor in South Asia [5]. In a 2019 survey of broiler veterinarians in the United States (US), coccidiosis (specifically *Eimeria maxima*) was ranked as the top disease-related issue in the opinion of the respondents [6]. A similar survey of the US Association of Veterinarians in Egg Production (AVEP) indicated that coccidiosis was considered the most important disease or condition in replacement layers reared cage-free, and the second most important in those reared in cages [6].

Several attempts have been made to quantify the consequences of coccidiosis in chickens. *Eimeria* lifecycles have been modelled for several parasite species, assessing rates of replication and/or associated pathology [7–12]. The financial cost has been estimated for countries including Ethiopia, India, Romania and the UK [4, 13–15], with a model published by Williams being most comprehensive and most widely referenced [16]. In the latter study, the annual cost of coccidiosis in chickens was estimated to exceed £38 m in the UK at 1995 prices. In the intervening period the UK chicken population has increased by 56% (1995–2018), with far larger expansions experienced in some other countries including Brazil, China and India, such that global chicken production is now double that of 25 years ago [2]. During this time, the Williams 1995 figure of £38 m has been extrapolated to provide estimates of the global cost of coccidiosis in chickens beginning at US$0.8 billion in 2002 [17], growing to US$2.4 billion in 2005 and US$3 billion in 2006 [1, 18], all using the original figures from 1995. In the same period the value of the UK £ sterling (GBP) has changed such that £1 in 1995 was equivalent to £1.94 in 2019 [19]. No further update has been published for the cost of coccidiosis in chickens and the calculation has not been repeated.

Poultry production has undergone considerable development over the last 25 years. Broiler growth rates have increased, feed conversion ratios (FCR) fallen and average days to market reduced in intensive systems [20]. Similarly, egg production has changed including longer, more productive laying cycles [21]. Poultry housing and management systems for intensively reared poultry have also evolved to improve health, welfare and productivity. Approaches to control of coccidiosis have also changed. In the Williams model description of 1995, only broiler breeders were assumed to receive anticoccidial vaccination [16], whereas vaccination using formulations of live wild-type (non-attenuated) or attenuated vaccines is now the dominant form of anticoccidial prophylaxis for layer replacement, layer and broiler breeding stocks in much of the World [22]. Vaccination is not yet so common in broiler production, although public and legislative pressures are encouraging the search for cost-effective alternatives to anticoccidial drugs, especially in countries such as the US, where (unlike the EU) ionophores are regulated as antibiotics. In response, 35–40% of US broiler companies have adopted annual cycles where two out of every six flocks receive anticoccidial vaccination instead of drugs [23]. More recently, it has been reported that more than 50% of US broilers are now raised antibiotic-free, indicating the absence of anticoccidial chemoprophylaxis [6]. In addition to vaccination as a replacement for chemoprophylaxis, bioshuttle programmes using vaccination with a wild-type (non-attenuated) product followed by an anticoccidial drug is increasingly popular in countries such as the US.

In this paper we describe the application of the Williams compartmentalised model to calculate the cost of coccidiosis in chickens using data collected in 2016 from countries representing six different continents, including updates to address recent changes in poultry husbandry and marketing. Understanding of the economic cost, or burden, of livestock diseases has evolved since the original publication [16, 24], recognising the contribution of indirect costs such as infrastructure and services as well as direct costs of control, mortality and morbidity. Thus, using the Williams model we have estimated the nominal financial cost of coccidiosis in chickens in 2016.

## Materials and methods

### Data collection

A questionnaire was prepared to capture data required to complete the ‘compartmentalised model for the estimation of the cost of coccidiosis’ described previously by Williams [16], including details of feed, drug and vaccine costs, health and performance parameters. The questionnaire was approved by the Social Science Research Ethical Review Board (SSRERB) of the Royal Veterinary College and assigned the reference URN SR2017-1248. Between three and ten industry representatives including veterinarians, farmers, integrators, poultry production and poultry health experts were surveyed in each of Brazil, Egypt, Guatemala, India, New Zealand, Nigeria, the United Kingdom (UK, including Great Britain & Northern Ireland) and the United States of America (US) between 2016 and 2017. Respondents were anonymised at the time of data collection. In the UK additional data were collected, anonymised and amalgamated by the British Poultry Council (BPC), producing a dataset that was representative of British poultry producers and integrators. Where a range of figures was recorded, the mid-point was calculated. International poultry production figures were accessed using FAOSTAT (http://www.fao.org/faostat/) [2], recording figures for 2016 to align with data collected from poultry industry representatives. FAOSTAT was originally accessed in 2018, but figures were subsequently updated by FAO and revised here as of May 19th, 2020. Additional details were accessed from Eurostat [25], accessed July 27th, 2020, and from broiler and layer management guides using the editions that were valid in 2016–2017, as referenced where relevant.

### Data analysis

The method developed by Williams [16] was adopted, including a series of 12 compartmentalised components covering costs related to chick purchase and rearing, performance, anticoccidial prophylaxis and therapy in broiler, layer and breeder chickens (Table 1). Equations used without modification are summarised, with supporting data presented for all modifications. Figures used in analysis were updated from data recorded in the UK alone in 1995 to Brazil, Egypt, Guatemala, India, New Zealand, Nigeria, the UK and the US in 2016–2017. The countries included were selected to represent major poultry producing regions in South America, North Africa, Central America, Asia, Oceania, sub-Saharan Africa, Europe and North America, respectively.

**Table 1:** Summary of compartments for estimation of the cost of coccidiosis and modifications undertaken here

Cost compartment (Williams [16])	Cost type	Modified	Modification
1. Prophylactic control for broilers	Control	Yes	Cost of vaccine, % broilers vaccinated
2. Vaccinating broiler breeders	Control	No	–
3. Therapeutic treatment for broilers against coccidiosis	Control	No	–
4. Therapeutic treatment for broiler breeders against coccidiosis	Control	No	–
5. Broiler mortality due to coccidiosis	Mortality	No	–
6. Reduced broiler weight due to coccidiosis	Morbidity	No	–
7. Increased FCR	Morbidity	No	–
8. Reduced egg production by broiler breeders	Morbidity	Yes	Hatchability
9. Prophylaxis during rearing of replacement layers	Control	Yes	Cost of vaccine, % replacement layers vaccinated
10. Prophylaxis for layer breeders	Control	Yes	Cost of vaccine, % layer breeders vaccinated
11. Therapeutic treatment of layer replacements during rearing	Control	No	–
12. Therapeutic treatment of layer breeders against coccidiosis	Control	No	–

### Prices, international currency and quantity calculations

The analysis used nominal prices from 2016. The currency exchange rates used in this study were set using Google Currency Converter on November 13^th^, 2016. Rates against one (1) UK £ (sterling) were 0.22 Brazilian real, 0.719 Egyptian pounds, 0.012 Indian rupees, 0.48 New Zealand dollars, 0.0021 Nigeria naira, and 0.719 US dollars (also used in Guatemala). US tons were converted to metric tonnes where necessary using the conversion factor 0.907. Calculated costs are presented in millions to a maximum of five significant figures to assist clarity.

## Results

### The cost of anticoccidial prophylaxis for commercial broiler chickens

Figures for annual production of broiler chickens can be accessed by country or region using FAOSTAT, presented as tonnes of dressed meat (search criteria: Production/Livestock primary/Region/production quantity/meat, chicken) or number slaughtered for meat (search criteria: Production/Livestock primary/Region/Producing animals slaughtered/meat, chicken), recognising that both will include a small proportion of non-broiler derived meat (e.g. spent hens) (Table 2). Consideration of the Cobb and Aviagen broiler management manuals suggested that 74.3% of a broiler carcass can be used for meat [26, 27], although the UK industry view was that 71% was more realistic, a marginal increase on the figure presented previously [16]. Thus, figures for dressed meat produced can be used to estimate total liveweight with a meat yield factor of 1.408 (Eqs. 1 and 2). For the UK, and other countries in the EU, accurate data regarding total liveweight can be accessed directly with no need for these calculations using Eurostat [25], indicating production of 1.79 million tonnes liveweight in the UK in 2016 (Table 2).

1 $${\text{Meat yield factor }} = { 1}00 / \, \% {\text{ carcass used}}$$

2 $${\text{Tonnes liveweight }} = {\text{ Tonnes dressed meat}} \times {\text{Meat yield factor}} .$$

**Table 2:** Values used to calculate the cost of anticoccidial prophylaxis in broiler and broiler breeder chickens

	UK-1995^a^	Brazil	Egypt	Guatemala	India	New Zealand	Nigeria	UK	USA
1. Cost of broiler prophylaxis/vaccination									
Tonnes dressed meat (millions)^b^	1.02	13.23	1.01	0.23	3.31	0.21	0.20	1.79^d^	18.71
% Carcass used	70.2	71.0	71.0	71.0	71.0	71.0	71.0	nr	71.0
FCR	1.98	1.72	1.6	1.56	1.65	1.47	1.7	1.6	1.75
% Formulated feed (i.e. not wheat/cereal)	95	85	85	85	80	100	80	90	86
% Starter	25	7.2	7.2	7.2	7.2	7.2	7.2	7.2	7.2
% Grower	55	31.8	31.8	31.8	31.8	31.8	31.8	31.8	31.8
% Finisher (inc. drug withdrawal)	20	61.0	61.0	61.0	61.0	61.0	61.0	61.0	61.0
Cost chemical/combination drug per tonne food	£2.74	£6.20	£0.72	£2.18	£0.72	£7.00	£7.35	£5.50	£5.95
Cost ionophore drug per tonne food	£2.97	£3.20	£2.41	£1.31	£1.44	£5.00	£3.15	£3.50	£2.44
*% BROILERS reared on drugs*	*ni*	*100*	*100*	*100*	*100*	*100*	*100*	*97*	*88*
Cost of in-feed drugs in starter diet (millions)	£1.88	£12.27	£0.10	£0.07	£0.32	£0.22	£0.21	£0.99	£14.90
Cost of in-feed drugs in grower diet (millions)	£4.48	£53.73	£1.48	£0.30	£1.41	£0.97	£0.91	£4.37	£65.83
Cost of in-feed drugs in finisher diet (millions)^c^	*ni*	£39.90	£2.13	£0.26	£4.05	£1.00	£0.56	£4.13	£44.12
*Vaccine (broiler) pence per dose*	*ni*	*1.3*	*1.15*	*0.49*	*3.0*	*1.54*	*4.2*	*3.0*	*0.49*
*% Broilers reared with vaccine*	*ni*	*0*	*0*	*0*	*0*	*0*	*0*	*3*	*12*
Cost of vaccination (millions)	*ni*	£0.00	£0.00	£0.00	£0.00	£0.00	£0.00	£0.94	£5.23
Number slaughtered per year (millions)^b^	624.8	5860.3	897.1	142.6	2411.6	110.0	202.8	1050.0	8908.9
Mean finishing weight broiler (Kg)	2.33	2.7	2	1.9	2.4	2.6	2.3	2.19	2.72
Total cost of broiler prophylaxis	£6.36	£105.8	£3.70	£0.63	£5.78	£2.19	£1.67	£10.44	£130.1
2. Cost of vaccinating broiler breeders									
% Broiler breeders in population	1.15	0.77	0.77	0.77	0.77	0.77	0.77	0.77	0.77
Vaccine (breeder) pence per dose	8	1.3	3	0.49	3	9.6	4.2	8	0.49
Total cost of broiler breeder prophylaxis (millions)	£0.57	£0.59	£0.21	£0.005	£0.56	£0.08	£0.06	£0.65	£0.33

Data collected from major poultry producing countries selected to represent South, Central and North America (Brazil, Guatemala, USA), North Africa (Egypt), sub-Saharan Africa (Nigeria), Asia (India), Europe (UK) and Oceania (New Zealand). Rows shown in italics represent new data related to anticoccidial vaccine use that were not included (*ni*) in the original model. ^a^Figures from the UK in 1995 used in the original study published by Williams [16]. ^b^Figures downloaded from FAOSTAT for the year 2016 (2020). ^c^Cost shown for 75% of actual period to account for average withdrawal period. ^d^Figure downloaded from Eurostat for the year 2016 (2020), representing total liveweight, not dressed meat. Thus, the meat yield factor was not required (nr).

Calculating the cost of anticoccidial prophylaxis using the Williams model for 1995 assumed that all broiler chickens received ionophore or non-ionophore (chemical) drugs [16]. By 2016, the situation was more complex. While the majority of broiler chickens continued to receive anticoccidial drugs in the UK, up to 3% of broilers may have been reared drug-free under organic or other systems [28]. The use of live anticoccidial vaccines in chicken production has increased significantly since 1995. Anticoccidial vaccination remains uncommon in UK broiler production, but 35–40% of US broiler producers used anticoccidial vaccines instead of chemoprophylaxis for at least two flocks per year by 2014 [23]. Here, for the UK calculation we assume that up to 3% of broiler chickens were vaccinated, and 97% of broilers received conventional chemoprophylaxis.

Anticoccidial prophylaxis for chickens using chemical or ionophore drugs is usually administered via the feed. Consideration of the feed conversion ratio (FCR) achieved during chicken production can be used to estimate total feed consumption. In the UK, our survey and the broiler management manuals suggested average FCR to have been 1.6 in 2016 [26, 27], although variation in efficiencies of production would be expected to change FCR, suggesting that the average may vary between producers. Thus, if 1.79 million tonnes liveweight chicken were produced in the UK in 2016, assuming an FCR of 1.6, the total quantity of chicken feed consumed would have been at least 2.87 million tonnes (Eq. 3).

3 $${\text{Feed consumption }} = {\text{ Tonnes liveweight}} \times {\text{ FCR}} .$$

Referring to the questionnaire, in the UK it is common practice to add an additional 10% whole wheat to the formulated ration, although the precise figure is likely to vary as the price of wheat fluctuates. Thus, formulated feed represented ~ 90% of the chicken feed consumed, equivalent to 2.58 million tonnes in 2016. Ross 308, followed by Cobb500 broiler chickens were the most popular lines used in the UK in 2016 so the Aviagen and Cobb broiler nutrition and performance supplements were considered for feed consumption parameters [26, 29]. The Aviagen figures indicate that ~ 6.2% of total feed would have been consumed as starter diet over the first 10 days for chicks on a 42 day programme, rising to 8.9% on a 35 day programme [26]. Here, we have assumed an intermediate 39 day programme, indicating that 7.2% of the 2.58 million tonnes formulated feed would have been consumed as starter diet (Table 2 and as above). While practices vary considerably between producers, assuming that a starter feed containing a chemical (i.e. non-ionophore), or more commonly a combination chemical + ionophore drug, was used at an average cost of £5.50 drug per tonne supplemented feed, the total cost of supplementation would have been £0.99 million for broilers on an intermediate 39 day programme in 2016 (Table 2, range £0.85-£1.22 million for 35 to 42 days). Between 27.3% and 39.0% of feed is consumed as grower diets from days 11 to 22 on 42 and 35 day broiler programmes, respectively, although many producers include additional switches during these periods. Ionophores are the most common anticoccidial drugs used in broiler production, representing 72.3% by weight of anticoccidial drugs sold in the UK in 2013, the last year that the Veterinary Medicines Directorate reported on ionophore use [30]. Assuming that ionophore anticoccidial drugs were used in combination products for the first grower phase on a 39 day programme, the total cost of supplementation would have been £4.37 million in 2016 based upon 31.8% of total feed consumed (Table 2; range £3.76–£5.37 million). The majority of feed is consumed in the finisher phase, from day 23 onwards, representing between 66.5% and 52.1% of formulated feed on 42 and 35 day programmes, respectively (61.0% for 39 days). Ionophores are commonly fed alone during this period at an average cost of £3.50 per tonne supplemented feed. Withdrawal periods vary from 0 to 5 days, depending on the product used, and were estimated to represent 25% of the finisher phase without chemoprophylaxis, indicating a total cost of supplementation of £4.13 million in 2016 (range £3.53–£4.50 million). Thus, the total estimated cost of anticoccidial-supplementation in feed was £9.50 million for those broilers that received chemoprophylaxis in 2016 (range £9.11–£10.12 million). Figures from FAOSTAT report that 1.05 billion chickens were reared for meat in the UK in 2016 (search criteria: Production/Livestock primary/Region/Producing animals slaughtered/meat, chicken). Assuming that 97% received anticoccidial chemoprophylaxis, the cost for each UK broiler chicken treated would have been £0.01 in 2016.

The cost of anticoccidial vaccination for broiler chickens is difficult to quantify, but based on our survey is approximately £0.03 per dose in the UK where live attenuated vaccines are licensed for use (Table 2). If 3% of the broiler chickens reared in 2016 were vaccinated, representing 31.5 million doses, the cost would have been £945,000. Thus, the total financial cost of prophylaxis for coccidiosis (anticoccidial drugs and vaccines) in the UK broiler sector is estimated to have been £10.44 million in 2016. Using the same equations populated with data collected from surveys in Brazil, Egypt, Guatemala, India, New Zealand, Nigeria and the US suggested that the annual costs of broiler prophylaxis using drugs or vaccines would have been £105.8, £3.70, £0.63, £5.78, £2.19, £1.67 and £130.1 million, respectively (Table 2).

### The cost of anticoccidial vaccination for broiler breeder chickens

As in 1995, broiler breeders are routinely vaccinated in the UK and much of the World against coccidiosis. In 1995 the percentage of broiler breeders used to produce commercial broiler progeny was estimated to have been at least 1.15% [16]. In 2016 this figure had dropped to 0.77%, equivalent to 130 chicks produced per broiler breeder hen, suggesting that 8.08 million breeding chickens would have been required to produce the 1.05 billion broilers reported from the UK in 2016. The average cost of anticoccidial vaccination per broiler breeder in the UK in 2016 was £0.08, unchanged from 1995, suggesting a total annual cost of £0.65 million (Table 2). Equivalent figures for Brazil, Egypt, Guatemala, India, New Zealand, Nigeria and the US would have been £0.59, £0.21, £0.005, £0.56, £0.08, £0.06 and £0.335 million, respectively, taking into account the variable costs per dose of the live wild-type or attenuated vaccines licensed for use in some of these countries. Vaccine used in Guatemala was provided from the US market, commonly delivered to chicks produced in the US and shipped to Guatemala.

### The cost of anticoccidial therapy for broilers and broiler breeders

The true occurrence of coccidiosis in broiler and broiler breeder stock is often poorly documented, representing commercially sensitive information. In the UK, the consensus opinion was that the occurrence of disease outbreaks was < 5% across all flocks, where assessment of ‘occurrence’ is usually based on gross pathology. Laboratory confirmation is less common. Here, we have used figures of 3% for broiler flocks, and 2% for broiler breeders (Table 3). The cost of therapeutic treatment per chicken was estimated to be £0.02 for broiler and broiler breeders. When broiler and broiler breeder chickens are treated for coccidiosis, therapy is administered to the entire house/pen, suggesting that 31.5 million broilers and 161,538 broiler breeders received treatment for coccidiosis in the UK in 2016 (Eq. 4, Table 3). Thus, the total cost of treatment for coccidiosis in the UK in 2016 was estimated to be £0.63 million for broilers, and £0.003 million for broiler breeders, although the true figures are likely to vary according to the actual occurrence of coccidiosis.

4 $${\text{Cost of therapy }} = {\text{ Number of chickens}} \times \, \% {\text{ Occurrence of coccidiosis }} \times {\text{ Cost of treatment}} .$$

**Table 3:** Values used to calculate the cost of anticoccidial therapy in broiler and broiler breeder chickens

	UK-1995^a^	Brazil	Egypt	Guatemala	India	New Zealand	Nigeria	UK	USA
3. Cost of broiler therapy against coccidiosis									
% Flocks affected by coccidiosis	2	8	35	80	5	80	30	3	10
Pence treatment per bird	3.18	3.10	0.43	7.19	1.20	0.2^b^	5.25	2.00	2.88
Total cost of broiler therapy (millions)	£0.40	£14.53	£1.35	£8.21	£1.45	£0.18	£3.19	£0.63	£25.64
4. Cost of broiler breeder therapy against coccidiosis									
% Flocks affected by coccidiosis	1	8	25	80	2	80	50	2	10
Pence treatment per bird	2.03	2.90	4.32	7.19	1.20	19.20	4.73	2.00	10.79
Total cost of broiler breeder therapy (millions)	£0.001	£0.10	£0.07	£0.06	£0.004	£0.13	£0.04	£0.003	£0.74

Data collected from major poultry producing countries selected to represent South, Central and North America (Brazil, Guatemala, USA), North Africa (Egypt), sub-Saharan Africa (Nigeria), Asia (India), Europe (UK) and Oceania (New Zealand). ^a^Figures from the UK in 1995 used in the original study published by Williams [16]. ^b^The cost of treatment in New Zealand was set artificially low since it is very rare for broilers to be treated for coccidiosis due to drug residue regulations.

Application of the same equation to costs in Brazil, Egypt, Guatemala, India, New Zealand, Nigeria and the US revealed considerable variation, underpinned by notably different estimates for the occurrence of coccidiosis from 5% to 80% for broilers, and 2% to 80% for broiler breeders (Table 3).

### The cost of broiler mortality caused by coccidiosis

Our survey suggested that approximately 2% of UK broilers would be expected to die or (more commonly) be culled in a house affected by coccidiosis (Table 4). Thus, if 31.5 million broilers were in houses affected by coccidiosis, mortality would have been 630,000 (Eq. 5). Note, the figure does not include losses due to subsequent mortality caused by other opportunistic pathogens.

**Table 4:** Values used to calculate the performance costs of coccidiosis in broiler and broiler breeder chickens

	UK-1995^a^	Brazil	Egypt	Guatemala	India	New Zealand	Nigeria	UK	USA
5. Cost of broiler mortality due to coccidiosis									
% Mortality due to coccidiosis	0.5	5.5	5.5	1.0	7.5	1.5	7.5	2.0	2.0
Value, including chick costs, at 3 weeks	£0.52	£0.62	£0.90	£0.65	£0.72	£0.77	£0.74	£0.80	£0.42
Av liveweight at slaughter (Kg)	2.33	2.7	2.0	1.9	2.4	2.6	2.3	2.19	2.72
Av slaughter price/Kg	£0.57	£1.25	£0.86	£0.52	£1.98	£0.96	£2.10	£0.80	£1.10
Cost of rearing bird to final weight (no chick costs)	£1.02	£0.95	£1.01	£0.79	£1.50	£0.62	£1.68	£1.12^b^	£0.36
Total cost of broiler mortality (millions)	£0.05	£78.52	£27.96	£0.96	£35.92	£1.14	£17.73	£0.90	£54.45
6. Cost of reduced weight due to coccidiosis									
Average broiler weight loss due to coccidiosis (Kg)^c^	0.1	0.1	0.1	0.1	0.1	0.1	0.1	0.1	0.1
Conservative prediction of weight loss (Kg)	0.05	0.07	0.07	0.07	0.07	0.07	0.07	0.07	0.07
Value loss per Kg (selling price per Kg)	£0.57	£1.25	£0.86	£0.52	£1.98	£0.96	£2.10	£0.80	£1.10
Total cost of reduced weight gain (millions)	£17.91	£512.8	£54.22	£5.17	£334.3	£7.39	£29.82	£58.80	£687.4
7. Cost of increased FCR									
Increased FCR as a consequence of coccidiosis^c^	0.1	0.1	0.1	0.1	0.1	0.1	0.1	0.1	0.1
Conservative estimate FCR increase^c^	0.05	0.05	0.05	0.05	0.05	0.05	0.05	0.05	0.05
Mean formulated feed price per tonne	£180.61	£280.00	£251.80	£393.00	£318.00	£297.60	£315.00	£275.00	£218.13
Mean wheat (or other cereal) price per tonne	£113.50	£140.00	£160.00	£178.00	£150.00	£192.00	£150.00	£160.00	£145.00
% formulated feed (i.e. not wheat/cereal)	95	85	85	85	80	100	80	90	86
Total cost of increased FCR (millions)	£13.15	£241.4	£16.88	£5.95	£66.26	£4.42	£4.03	£23.60	£273.9
8. Cost reduced egg production by broiler breeders									
% Broiler breeders that were parent hens	72.4	90.9	90.9	90.9	90.9	90.9	90.9	90.9	90.9
*% Broiler egg hatchability*	*ni*	*85*	*85*	*85*	*85*	*85*	*85*	*85*	*85*
Cost one day old broiler chick	£0.23	£0.30	£0.22	£0.42	£0.34	£0.34	£0.38	£0.33	£0.23
Cost of lower broiler breeder egg production (millions)	£0.01	£0.84	£0.29	£0.28	£0.10	£0.18	£0.23	£0.04	£1.22

Data collected from major poultry producing countries selected to represent South, Central and North America (Brazil, Guatemala, USA), North Africa (Egypt), sub-Saharan Africa (Nigeria), Asia (India), Europe (UK) and Oceania (New Zealand). Rows shown in italics represent new data related to hatchability that were not included (*ni*) in the original model. ^a^Figures from the UK in 1995 used in the original study published by Williams [16]. ^b^Figures taken from the Cobb broiler management guide (Cobb-Vantress [29]). ^c^Figures not available; replaced with those from Williams [16].

5 $${\text{Mortality due to coccidiosis }} = {\text{ Number chickens affected by coccidiosis}} \times \, \% {\text{ Mortality}} .$$

Losses attributed to mortality were divided in the Williams model into the chicken value, including cost of purchase (Eq. 6), and the revenue that had been lost when chickens were not sold minus the rearing costs that were saved due to premature mortality (Eq. 7) [16]. In total 630,000 broilers were estimated to have died due to coccidiosis in the UK in 2016 (as above). In the model, chick value was calculated at an average time point of 3 weeks of age, representing the beginning of the period when mortality is most likely to occur [31]. From our survey, each individual broiler would be worth on average £0.80 at 3 weeks of age, indicating a total cost of £0.50 million (Eq. 6). The average liveweight for a commercial broiler in the UK at the time of slaughter in 2016 was 2.191 kg [27]. From our survey, an average of £0.80 was paid per Kg at the time of slaughter, indicating a loss of £1.75 per individual, balanced by a reduction in rearing costs of £1.12 per chicken lost, resulting in a net loss of £0.63 per chicken or £0.40 million in total (Eq. 7). Thus, the total cost of broiler mortality in the UK in 2016 was £0.90 million. The equivalent costs for Brazil, Egypt, Guatemala, India, New Zealand, Nigeria and the US would have been £78.52, £27.96, £0.96, £35.92, £1.14, £17.73 and £54.45 million, respectively (Table 4).

6 $${\text{Value of chickens lost due to coccidiosis }} = {\text{ Chicken mortality}} \times {\text{ Estimated value at 3 weeks}}$$

7 $${\text{Net loss }} = \, [({\text{Number dead}} \times {\text{ Av}}.{\text{ liveweight at slaughter}}) \, \times \, \pounds{\text{ per Kg at slaughter}}] \, {-} \, \left( {{\text{Number dead }} \times {\text{ Cost of rearing to final weight from 3 weeks}}} \right).$$

Assuming a lower level of mortality in broiler breeder flocks given that most will have been vaccinated, the number of broiler breeders lost due to coccidiosis per annum would have been relatively small. The cost of broiler breeder loss was not calculated in the original Williams model for this reason, and because the actual cost of breeding stock is very difficult to define.

### The cost of morbidity: reduced broiler weight gain due to coccidiosis

*Eimeria* are ubiquitous and it is likely that most broiler chickens are exposed to one or more *Eimeria* species during their lives [32]. While severe coccidiosis can result in mortality, morbidity is far more common with both malabsorptive and haemorrhagic enteric disease compromising nutrient absorption and body weight gain [33]. In the original model the average effect of coccidiosis was estimated to reduce final bodyweight by 0.1 kg when compared to an unexposed equivalent, although 0.05 kg was used as a conservative estimate [16]. Consideration of more recent studies with malabsorptive *Eimeria* species such as *E. acervulina* and *E. maxima* suggest a greater impact, with reductions in excess of 0.1 kg [33, 34], although the consequences of infection with less pathogenic species such as *E. mitis* and *E. praecox* are less clear. In line with these data, and recognition of increased growth rate in modern broiler chickens and hence greater likely impact of infection, our conservative estimate of final reduction in live bodyweight as a consequence of *Eimeria* infection was raised to 0.07 kg (Table 4). Thus, with 1.05 billion broiler chickens produced in the UK in 2016, the predicted total loss of body weight caused by *Eimeria* infection would have been 73,500,000 kg. Given the value of £0.80 per Kg bodyweight at slaughter (as above), the loss would have equated to a total of £58.80 million (Eq. 8, Table 4). Changing the estimated reduction in live bodyweight by ± 0.02 kg resulted in a 28.6% increase/decrease in total cost (between £42.00 and £75.60 million). The equivalent costs of a 0.07 kg reduction in broiler body weight gain for Brazil, Egypt, Guatemala, India, New Zealand, Nigeria and the US would have been £512.78, £54.22, £5.17, £334.26, £7.39, £29.82 and £687.43 million, respectively.

8 $${\text{Cost of reduced weight gain }} = \, \left( {{\text{Number of chickens}} \times {\text{ Predicted weight loss }}\left( {\text{Kg}} \right)} \right) \, \times \, \pounds{\text{ per Kg at slaughter}} .$$

### The cost of morbidity: higher feed conversion ratios (FCR) due to coccidiosis

In addition to lower final bodyweight, reduced nutrient absorption as a consequence of coccidiosis will also be reflected by a higher feed conversion ratio (FCR). A higher FCR illustrates the requirement for greater feed intake to achieve the same bodyweight gain due to inefficiencies in diet utilisation and in additional nutrient partition to repair damaged enteric tissues and the birds immune response. The influence of *Eimeria* infection on FCR is difficult to define. A wide range of variation has been reported, often following significant parasite challenge that might exceed common levels of challenge in the field. In the original model, Williams considered the effect of *Eimeria* infection on FCR to be an increase of 0.1, using 0.05 as a conservative measure. We have used the same figures here (Table 4). From our survey, broiler diets commonly include 10% wheat at an average cost of £160 per tonne in the UK in 2016, although wheat prices can vary significantly resulting in notable variation over time. Formulated feed, commonly representing 90% of the total diet, cost on average £275 per tonne (Table 4). Combined, the mean feed price can be calculated using Eq. 9 to be £263.50 per tonne in the UK in 2016. Thus, using the figures for broiler liveweight produced per annum described above, a predicted FCR increase of 0.05, and a mean feed price of £263.50 per tonne, the total cost of increased FCR due to coccidiosis was £23.60 (Eq. 10). Changing the estimated increase in FCR by ± 0.02 resulted in a 40.0% increase/decrease in total cost (between £14.16 and £33.03 million). Feed costs and ratios for formulated: non-formulated feed use varied between countries (Table 4), resulting in total costs of £241.40, £16.88, £5.95, £66.26, £4.42, £4.03 and £273.89 million for Brazil, Egypt, Guatemala, India, New Zealand, Nigeria and the US, respectively, when using an increased FCR of 0.05.

9 $${\text{Mean feed price }} = \, \left( {{\text{Mean price of formulated feed }} \times \, \% {\text{ formulated feed used}}} \right) \, + \, \left( {{\text{Mean price of non}} - {\text{formulated feed }}\left( {\text{cereal}} \right) \, \times \, \% {\text{ non}} - {\text{formulated feed used}}} \right)$$

10 $${\text{Cost of increased FCR }} = \, \left( {{\text{Broiler liveweight produced }}\left( {{\text {Kg}},{\text{ from Eq. 2}}} \right) \times {\text{ Predicted FCR increase}}}\right) \, \times {\text{ Average total feed cost }} \left( {{\text{Kg}},{\text{ Eq. 9}}} \right).$$

### The cost of morbidity: lower broiler breeder egg production

In the UK in 2016, we already estimated 161,538 broiler breeder chickens would have been affected by coccidiosis. A sex ratio of one male per ten females has been described for modern broiler breeder chickens [35], suggesting that 146,853 broiler breeder hens were used. The effect of *Eimeria* infection on egg production is not clear. The Williams model took a conservative approach, suggesting the loss of one egg per hen, per year. An additional parameter not considered in the original model is hatchability. While one egg may have been lost per hen, per year, only 85% might have been expected to yield a chick [36]. From our survey, the average cost for a day old broiler chick in the UK was £0.33, suggesting a total loss of £0.04 million. The equivalent costs for Brazil, Egypt, Guatemala, India, New Zealand, Nigeria and the US were £0.84, £0.29, £0.28, £0.10, £0.18, £0.23 and £1.22 million, respectively (Table 4), with the differences reflecting different levels of coccidiosis reported in broiler breeder flocks from different countries.

### The cost of anticoccidial prophylaxis for commercial layer chicken replacements

The number of layers reared in the UK each year can be estimated using FAOSTAT (search criteria: Production/Livestock primary/Region/Producing animals/slaughtered/eggs, hen, in shell), indicating that 53,489,000 hens were reared in total. In the Williams model, it was important to differentiate between replacement hens kept in cages or on litter, since different anticoccidial prophylaxis programmes were used. However, comparison of management guides for chickens reared in enriched cages or on the floor indicate that both now follow step-down programmes [37, 38]. Figures released by the Royal Society for the Prevention of Cruelty to Animals (RSPCA) suggest that 48% were accommodated as laying stock in enriched cages, with 1% in barn systems and 51% free range, of which 2% were organic [39] (Table 5).

**Table 5:** Values used to calculate the cost of prophylaxis and therapy in layer and layer-breeder chickens

	UK-1995^a^	Brazil	Egypt	Guatemala	India	New Zealand	Nigeria	UK	USA
9. Cost prophylaxis during rearing of replacement layers									
Number layer chickens (millions)^b^	32.0	329.0	34.2	20.1	382.7	3.7	111.4	53.5	365.3
% Reared in cages	60	70	65	10	80	50	10	48	50
% Reared on litter or ground (barn, free-range)	40	30	35	90	20	50	90	52	50
*Vaccine (layer) pence per dose*	*ni*	*1.3*	*1.15*	*0.49*	*3*	*9.6*	*4.2*	*8*	*0.49*
Feed consumed during step-down (Kg, weeks 0–3)^c^	0.36	0.40	0.40	0.40	0.40	0.40	0.40	0.40	0.40
Feed consumed during step-down (Kg, weeks 4–6)^c^	0.74	0.71	0.71	0.71	0.71	0.71	0.71	0.71	0.71
Feed consumed during step-down (Kg, weeks 7–12)^c^	2.00	2.22	2.22	2.22	2.22	2.22	2.22	2.22	2.22
*% Reared fed drugs*	*ni*	*30*	*5*	*5*	*90*	*5*	*95*	*5*	*5*
*% Reared vaccinated*	*ni*	*70*	*95*	*95*	*10*	*95*	*5*	*95*	*95*
Total cost of layer replacement prophylaxis (millions)	£0.11	£3.87	£0.38	£0.10	£2.53	£0.34	£1.15	£4.09	£1.82
10. Cost prophylaxis for layer breeders									
% Layer flock as breeders^d^	1.21	1.21	1.21	1.21	1.21	1.21	1.21	1.21	1.21
Vaccine (breeder) pence per dose	*ni*	*1.3*	*1.15*	*0.49*	*3*	*9.6*	*4.2*	*8*	*0.49*
*% Layer breeders on drugs*	*ni*	*10*	*0*	*0*	*50*	*0*	*90*	*0*	*0*
*% Layer breeders vaccinated*	*ni*	*90*	*100*	*100*	*50*	*100*	*10*	*100*	*100*
Total cost of layer breeder prophylaxis (millions)	£0.003	£0.05	£0.005	£0.001	£0.11	£0.004	£0.02	£0.05	£0.02
11. Cost of treating layer replacements during rearing									
% Flocks affected by coccidiosis	5	5	10	80	5	80	15	4	5
Pence total cost of treatment	1.9	2.9	4.32	7.19	1.2	19.2	4.7	2.0	2.5
Total cost of layer replacement therapy (millions)	£0.01	£0.14	£0.05	£0.92	£0.05	£0.28	£0.71	£0.02	£0.23
12. Cost of treating layer breeders during rearing									
% Flocks affected by coccidiosis	5	5	10	80	5	80	15	4	5
Pence total cost of treatment	1.9	2.9	4.32	7.19	1.2	19.2	4.7	2.0	2.5
Total cost of layer breeder therapy (millions)	£0.0004	£0.006	£0.002	£0.01	£0.003	£0.007	£0.009	£0.0005	£0.005

Data collected from major poultry producing countries selected to represent South, Central and North America (Brazil, Guatemala, USA), North Africa (Egypt), sub-Saharan Africa (Nigeria), Asia (India), Europe (UK) and Oceania (New Zealand). Rows shown in italics represent new data related to anticoccidial vaccine use that were not included (*ni*) in the original model. ^a^Figures from the UK in 1995 used in the original study published by Williams [16]. ^b^Figures downloaded from FAOSTAT for the year 2016 (2020). ^c^Figures taken from the Hy-Line commercial layer management guide (Hy-Line, 2016a). ^d^Figures not available; replaced with those from Williams [16].

In the original Williams model the use of vaccination in anticoccidial prophylaxis for layer chickens was not considered, reporting 100% chemoprophylaxis [16]. By 2016, the situation had changed significantly, with at least 95% of layer chickens receiving live anticoccidial vaccination in the UK. From our survey, the average cost of an anticoccidial vaccine was 8p per dose in UK in 2016 (Table 5). Thus, if 95% of laying hens received vaccines, the total cost would have been £4.06 million.

Comparison of management guides for rearing commercial layer chickens in caged or alternative systems in UK suggested that stock reared using anticoccidial chemoprophylaxis usually follow step-down approaches divided into two or three different phases [37, 38]. Most commonly, ionophores were included at 100% of the optimal recommended rate from 0 to 6 weeks, or 0 to 3 and 4 to 6 weeks of age, respectively, followed by a final phase with ionophore at the minimum registered inclusion rate for 7–12 weeks [16]. The management guide suggests consumption of 0.40 kg, 0.71 kg and 2.22 kg feed during each phase of the three step system, respectively [38] (Table 5). Using the cost of £3.50 per tonne for ionophore supplementation at 100% of the recommended rate (as described above), the total cost of anticoccidial chemoprophylaxis for replacement layer hens in UK in 2016 was £0.03 million.

When the costs of prophylaxis for replacement layer chickens using drugs and vaccines are combined the total cost in 2016 in the UK would have been £4.09 million. Using the figures collected from our surveys in Brazil, Egypt, Guatemala, India, New Zealand, Nigeria and the US the total cost of prophylaxis for replacement layer hens was £3.87, £0.38, £0.10, £2.53, £0.34, £1.15 and £1.82 million, respectively, in 2016 (Table 5).

### The cost of anticoccidial prophylaxis for layer breeder chickens

The majority of layer breeder chickens are now routinely vaccinated in the UK, and much of the World, to control coccidiosis. Assuming that the ratio of layer breeders to commercial layer progeny remained comparable to that used for 1995 (1.21%, Table 5), 647,217 layer breeder chickens would have been required in the UK in 2016. The average cost of anticoccidial vaccination per layer breeder in the UK in 2016 was 8p. Assuming a ratio of vaccination to chemoprophylaxis comparable to that used for replacement layers, the total annual cost of layer breeder prophylaxis would have been £0.05 million in the UK (Table 5). Equivalent figures for Brazil, Egypt, Guatemala, India, New Zealand, Nigeria and the US would have been £0.05, £0.005, £0.001, £0.11, £0.004, £0.02 and £0.02 million, respectively, again influenced by a far lower vaccine cost per dose in the US.

### The cost of anticoccidial therapy for layer and layer breeder chickens

Based upon the figures shown above, 27,814,280 layer and 647,217 layer breeder chickens may have been floor-raised in UK in 2016. If ~ 4% of flocks required treatment for coccidiosis at a cost of 2p per individual (Table 5), the total cost would have been £0.02 and £0.0005, respectively (using Eq. 4). When considering costs for Brazil, Egypt, Guatemala, India, New Zealand, Nigeria and the US the figures demonstrated notable variation, reflecting differences in the occurrence of coccidiosis and the costs of therapy (Table 5).

### The financial cost of coccidiosis in chickens

Combined, the 12 compartments previously described in the Williams model have been used to estimate the cost of coccidiosis in the UK [16]. Repeating the analysis using figures for the UK updated to 2016 suggest a combined total nominal financial cost of £99.23 million per annum; a 2.6-fold increase from the last calculation in 1995.

Considerable interest exists in calculating the global cost of coccidiosis in chickens. Extrapolation of any sort is hazardous and will certainly be inaccurate, failing to adapt to regional variation in consumer preferences, markets, environments, husbandry and biosecurity. In an effort to reduce such inaccuracies we have also calculated the total cost of coccidiosis using an updated version of the Williams model for Brazil, Egypt, India, Guatemala, New Zealand, Nigeria and the US, each representing a different continent or distinct region, and presenting a range of costs between £16.34 and £1175.88 million (Table 6). Taking these countries as exemplars, the total cost of coccidiosis in chickens in 2016 per continent or region was shown to vary between £112.39 and £5181.97 million (Table 6), resulting in a global estimated total cost of £10,362.03 in 2016. Adjusting the estimates used for weight gain lost during infection and FCR increase as a consequence of infection by ± 0.02 in each country resulted in between 17.3% and 26.4% variation (Table 6). Thus, the range for the global cost of coccidiosis was estimated to fall between £7711.51 and £13,012.54 million per annum. Based upon the number of chickens slaughtered and the total calculated cost, the average cost of coccidiosis per chicken produced was £0.16.

**Table 6:** The total cost of coccidiosis calculated per country and extrapolated per region

Region	Slaughtered (millions)^a^	Example country	Total cost of coccidiosis (millions £)		Range^b^
		(% slaughtered per region)	Example country	Extrapolated to region	
N. Africa	1971.7	Egypt (45.5%)	£105.13	£231.04	±26.4%
Sub-Saharan Africa	2409.4	Nigeria (8.4%)	£58.67	£696.82	±27.3%
Asia	27 957.7	India (8.6%)	£447.01	£5181.97	±17.3%
Europe	10 831.5	UK (9.7%)	£99.23	£1023.58	±25.4%
N. America	9615.6	USA (92.7%)	£1175.88	£1269.14	±17.3%
C. America	2297.7	Guatemala (6.2%)	£22.31	£359.46	±23.8%
S. America	9094.2	Brazil (64.4%)	£958.62	£1487.61	±26.0%
Oceania	757.0	New Zealand (14.5%)	£16.34	£112.39	±21.2%
World	65 326.8			£10 362.03	± 25.6%

^a^ Figures downloaded from FAOSTAT for the year 2016 (2020). ^b^The range is represented by the percentage change incurred when adjusting estimated body weight gain lost and FCR increased by +0.02 (higher impact) or − 0.02 (lower impact).

Partition of the financial cost calculated for each country into costs associated with control (drugs for prophylaxis, vaccines), mortality or morbidity revealed notable variation (Table 7). The contribution to cost attributed to control was greatest in Guatemala (44.6%) and lowest in India (2.4%). Costs associated with mortality were proportionately highest in Nigeria (30.2%) and Egypt (26.2%), lowest in the UK (0.9%). The costs of morbidity were most important, representing more than 80% of the total financial cost in India, the UK and the US (Table 7).

**Table 7:** The contribution of costs for control, mortality and morbidity to the cost of coccidiosis (millions)

Country	Cost of control (% total)	Cost of mortality (% total)	Cost of morbidity (% total)
Brazil	£125.09 (13.0)	£78.52 (8.2)	£755.01 (78.8)
Egypt	£5.79 (5.5)	£27.96 (26.6)	£71.39 (67.9)
Guatemala	£9.95 (44.6)	£0.96 (4.3)	£11.40 (51.1)
India	£10.47 (2.4)	£35.92 (8.0)	£400.61 (89.6)
New Zealand	£3.21 (19.6)	£1.14 (7.0)	£11.99 (73.4)
Nigeria	£6.86 (11.7)	£17.73 (30.2)	£34.07 (58.1)
UK	£15.89 (16.0)	£0.90 (0.9)	£82.44 (83.1)
US	£158.88 (13.5)	£54.45 (4.6)	£962.55 (81.9)

## Discussion

Understanding the financial cost of diseases that compromise animal production and welfare is important, providing a baseline for comparison of husbandry systems, risk factors and interventions [40]. *Eimeria* are the most economically significant parasites of poultry but their true cost to producers remains unclear. In 1995, the cost of coccidiosis to UK chicken production was estimated to be £38.59 million, of which 98.1% was attributed to the broiler sector. Recalculating the figure using prices from 2016, including costs associated with vaccination, indicate a financial cost of £99.23 million per annum, 95.1% of which derived from broiler production. The notable increase in cost can be explained in part by the larger chicken population and currency inflation, although costs associated with broiler prophylaxis and impacts on growth (reduced weight gain and increased FCR) were notably higher, likely amplified by the considerable genetic progress achieved by the primary breeding companies in selection for faster growing and more feed-efficient broiler stock [20]. The increased cost of coccidiosis may also have been influenced by the withdrawal of antibiotic growth promoters (AGPs) from chicken diets. The impact of coccidiosis on gut integrity can be more severe in chickens that are not receiving AGPs as the damage caused by coccidiosis provides substrates for the replication of certain bacteria, disrupting the balance of the intestinal microbiome [41, 42], potentially exacerbating malabsorption and enteritis, decreasing growth rates and increasing FCR.

The number of chickens estimated to be affected by coccidiosis was also notably higher than the figures used by Bennett and Ijpelaar, who predicted in 2005 a range of costs in the UK between £10.2 and £14.2 million per annum [4]. The percentage of broiler chickens lost due to coccidiosis was notably higher in every country surveyed in 2016 than the UK in 1995, possibly representing an underestimate in the original model [16]. Costs attributed to coccidiosis in layer replacements in the UK remained a small proportion of the total (4.15% of the total cost in 2016), but represented a 37-fold increase from 1995. The increase was primarily due to greater costs of prophylaxis, reflecting the switch from chemoprophylaxis to vaccination driven in part by the BEIC Lion code that forbids the use of in-feed anticoccidials from the age of 12 weeks in replacement layers [43]. Additionally, the use of relatively expensive live attenuated anticoccidial vaccines in the UK that include most or all *Eimeria* species will have increased the cost further. It is worth noting that levels of vaccination in replacement layer stock may vary between countries. The increase was replicated in layer breeding stock.

Application of the updated model to data collected from surveys in Brazil, Egypt, Guatemala, India, New Zealand, Nigeria and the US revealed considerable variation in the costs of production, the occurrence of infection and the outcomes of disease. The occurrence of coccidiosis was considered to be far higher in Egypt, Guatemala, New Zealand and Nigeria than the other countries surveyed, directly contributing to increased costs for treatment and mortality. The visible consequences of clinical coccidiosis were lower in Brazil, India, the UK and the US, where costs associated with morbidity were proportionately higher. The balance between losses incurred due to coccidiosis and the costs of prevention may reflect country-specific access to best practice technologies and chicken lines that are best adapted to their environment(s). Variation in the ongoing development of capacity and practice in each country is likely to have contributed to the differences reported in mortality and is not directly captured in this model.

The prices of feed, and especially vaccine doses, were far lower in the US than any of the other countries, limiting the costs of prophylaxis per layer and broiler/layer breeder, and the consequences of increased FCR. Countries such as the US routinely use non-attenuated anticoccidial vaccines with a greater productive capacity, and thus lower cost, than the attenuated vaccines licenced for use in the EU. It is worth noting that bioshuttle programmes, including use of a non-attenuated live vaccine followed by an anticoccidial drug, are increasingly popular in countries such as the US, incurring increased costs due to tandem prophylaxis and suggesting that figures for the US are an underestimate. The cost of coccidiosis in chickens in India has previously been estimated to be 1.14 billion Indian rupees (INR) in 2003–2004 [13], equivalent to £17 million, based upon a historic conversion rate of £1: INR 67 [44]. The figure presented here is far higher, in part reflecting the rapid expansion of poultry production in the region [3], but perhaps also a more comprehensive model. Previous figures for the other countries assessed were not available to be compared. Flock-level analyses have been reported from Ethiopia and Romania, identifying costs of 898.80–5301.80 Ethiopia Birr per farm, or 0.55 and 0.53 Birr per chicken in small and large scale farms, respectively, in Ethiopia [15], and of EU€3162.4 per flock in Romania in 2010 [14]. Fornace and colleagues assessed the impact of individual *Eimeria* species occurrence on the viability of small broiler farms in Ghana, Tanzania and Zambia, calculating farm gross economic margins and identifying reductions associated with more pathogenic species [45]. The impact of sub-clinical *Eimeria* infection has been investigated in Norway, where the authors identified a significant impact but did not discuss costs [46].

Figures used to define the ‘global cost’ of coccidiosis in chickens have been cited widely and have varied enormously [1, 17, 18]. We have estimated the financial cost for eight key countries, across six continents, which are defined by a significant contribution to chicken production in their region. Brazil and the US, selected to represent South and North America, are among the World’s biggest producers of poultry. Egypt and Nigeria, chosen to represent North and sub-Saharan Africa, lead production within their respective regions. India is host to one of the biggest expansions in poultry production in the World [3]. Guatemala and New Zealand were pragmatic selections based on availability of data. While production levels are far lower in the latter countries, they are representative of their wider regions. Using data from these countries and the UK, representing Europe, we have estimated the total financial cost of coccidiosis in chickens per region in an effort to reduce variation. It is important to note that the figures are far from comprehensive and doubtless miss regional variation from other countries. Nonetheless, extrapolating by continent or region, rather than from a single country, does serve to reduce gross variation and strengthen a tentative estimate of the global cost of coccidiosis in chickens. It is important to note that this estimate remains based on a model that relies on many assumptions. As such, the figure of ~ £10.36 billion, or £0.16 per chicken produced, can only be considered to be a guide. Variation of important parameters such as body weight gain lost in Kg and FCR (both ± 0.02) produced a range of estimates from £7.71 to £13.01 billion per annum. Variation in other parameters would expand the range further. The model does not take an entirely holistic view, omitting details such as costs of rent, loans or mortgages, overheads such as electricity and water, or depreciation of facilities and equipment, all of which may serve to increase the true economic cost. Similarly, the model does not include the consequences of gut dysbiosis caused by coccidial infection. *Eimeria* infection has been shown to modify enteric bacterial population structures with significant variation in commensal genera such as *Bacteroides* and *Lactobacillus* [41, 47]. Variation in microbiome composition has been associated with higher or lower FCR [48], potentially exacerbating the two biggest components of the cost of coccidiosis (reduced weight gain and increased FCR). *Eimeria* infection has also been associated with increased pathogen carriage. Concurrent *E. tenella* infection can increase intestinal *Campylobacter jejuni* and *Salmonella enterica* Typhimurium load [42, 49]. Most importantly, *Eimeria* co-infection is a major contributory factor to necrotic enteritis (NE) caused by *Clostridium perfringens* [50]. The global cost of NE has been estimated to exceed US$6 billion [51], adding considerable extra indirect expense to the direct cost of coccidiosis in chickens. The influence of gut dysbiosis due to *Eimeria* infection on litter quality has also not been included. Wet litter resulting from dysbiosis is a notable risk factor for pododermatitis, a leading cause of ill health, culling, condemnation and quality downgrades [52]. Pododermatitis is also used as a welfare indicator; its occurrence can result in reputational damage to a producer/integrator. Practically, wet litter caused by dysbiosis can incur extra costs associated with additional bedding, labour and electricity for extraction fans and heating to remove moisture. In the future, understanding the broader contribution of *Eimeria* infection and coccidiosis to overall health burdens in chickens will underpin improved estimates of true economic cost beyond the absolute figures provided here. Projects such as the Global Burden of Animal Diseases (GBADs) aim to explore this topic, establishing baseline figures for production of livestock in the absence of pathogens and defining the consequences of infection [40].

The most significant addition required to update the Williams model to calculate the cost of coccidiosis in chickens related to the far wider use of live anticoccidial vaccines. Anticoccidial vaccines have become the dominant form of anticoccidial prophylaxis in layer and breeding stock around much of the World [22], with burgeoning interest in “no antibiotics, ever” food production expected to increase vaccine use further. It has been suggested that more than 40% of US broiler producers now use anticoccidial vaccines in at least one flock each year [53], supported by statistics from a leading industry benchmark that more than 30% of commercial broilers sold in the US since 2016 have received an anticoccidial vaccine (personal communication). Expanding the use of live vaccines further has been limited by production capacity, especially for the less productive attenuated vaccines used in the EU [54]. The possible future emergence of recombinant or vectored anticoccidial vaccines would require further amendments to the model and could significantly influence the economics of chicken production.

In conclusion, the global cost of coccidiosis in chickens is estimated to have been ~ £10.36 billion in 2016, including losses during production and costs for prophylaxis and treatment. As the human population continues to expand and the challenge to achieve food security increases [3], improving understanding and control of economically significant pathogens of livestock remains essential.

## Data Availability

The datasets supporting the conclusions of this article are included within the article.

## Abbreviations

AGPs: antibiotic growth promoters
AVEP: Association of Veterinarians in Egg Production
BBSRC: Biotechnology and Biological Sciences Research Council
BPC: British Poultry Council
EC: European Commission
EU: European Union
FCR: feed Conversion Ratio
GBADS: global Burden of Animal Diseases
INR: Indian rupees
NE: necrotic enteritis
RSPCA: Royal Society for the Prevention of Cruelty to Animals
SSRERB: Social Science Research Ethical Review Board
UK: United Kingdom
US: United States of America
